# Achieving Prudent Dementia Care (*Palliare*): An International Policy and Practice Imperative

**DOI:** 10.5334/ijic.2497

**Published:** 2016-12-31

**Authors:** Debbie Tolson, Anne Fleming, Elizabeth Hanson, Wilson de Abreu, Manuel Lillo Crespo, Rhoda Macrae, Graham Jackson, Simona Hvalič-Touzery, Iva Holmerová, Pirkko Routasalo

**Affiliations:** Alzheimer Scotland Centre for Policy and Practice, Hamilton, The University of the West of Scotland, ML3 0JB, UK; Bishopbriggs, Glasgow, UK; Nationellt kompetenscentrum Anhöriga, Box 762, 391 27 Kalmar, Sweden; Porto School of Nursing, Rua Dr Antonio Bernardino Almeida, Porto, Portugal; University of Alicante, Campus de San Vicente Raspeig s/n, Alicante, Spain; Faculty of Health Care Jesenice, Dean’s Office, Spodnji Plavž 3, SI-4270 Jesenice, Slovenia; Gerontolgicke centrum, Simunkova 1600, 182 000 Praha 8 – Kobylisy, Czech Republic; Turku University of Applied Sciences Ltd TUAS, Joukahaisenkatu 3A, Finland

**Keywords:** advanced dementia, Prudent Healthcare, integrated care, workforce

## Abstract

This paper examines the provision of integrated advanced dementia care within seven European countries and critically reviews the potential contribution of the Prudent Healthcare perspective as a starting point for reform. Progressive efforts to innovate, promote quality and integrate care are tempered with the reality of resource constraints. Some policy makers in Europe and North America have turned their attention to the principles of Prudent Healthcare as a potential mechanism to maximise benefits for patients within available resources. As dementia progresses, living well requires increasing levels of support and care, people living with advanced dementia have complex health and social care needs, are highly dependent on others but are not yet at the terminal end stage of the condition. People with advanced dementia can benefit from a dementia specific palliative approach to care (*Palliare*), that helps them to live the best life possible for the months and often years they live with advanced dementia. It is also highly desirable to align policy innovations with integrated palliative care practice models and the education of the dementia workforce to accelerate informed improvements in advanced dementia care. There may be some coherence, at least superficially between Prudent Healthcare and integrated palliative care models such as *Palliare*. It is argued that for successful implementation, both require practitioners to be equipped with knowledge and skills and be empowered to deliver high quality care often within impoverished care environments. Adoption of the prudent perspective will however require development of a repertoire of approaches to hear the voice or proxy voice of people living with advanced dementia and to commit to the development and implementation of new evidence for advanced dementia practice. Evidence informing this policy debate draws upon contemporary literature and policy and the findings from research activities undertaken by the *Palliare* project supported through the Erasmus+ K2 Strategic Partnerships funding programme.

## Introduction

Dementia is a major public health concern, in Europe the number of people with dementia is predicted to increase from 9.95 million in 2010 to 18.65 million by 2050 [1,2]. The swift increase in the number of people with a diagnosis of dementia has prompted calls for countries across the globe to develop national actions plans and for common guidelines to inform practitioner training [3]. Adding to the change momentum, the G8 Dementia Summit in 2013 recommended that countries collaborate to build solutions and service models across the continuum of care [4].

Progressive efforts to innovate, promote quality and integrate care are tempered with the reality of resource constraints and the complexity of transformational change. Accordingly some policy makers in Europe and North America are turning their attention to the principles of Prudent Healthcare as a potential mechanism to ensure that we maximise benefit for patients within available resource [5]. The Prudent Healthcare paradigm is used to examine the delicate balancing act between effective and efficient policy making and policy implementation in relation to dementia care. In particular the focus is on advanced dementia, a point in the care experience where individuals are living with complex health and social care needs, are highly dependent on others but are not yet at the terminal end stage of the condition, and would benefit from an approach to care that helps them to live the best life possible. This approach will be referred to as *‘Palliare’* [6]. An important feature of *Palliare* is tailoring therapeutic interventions to the individuals’ health needs and achieving balance in terms of wider aspects of care and compassion for a progressive and complex condition. These complex physical healthcare needs must be addressed in tandem with psychosocial and spiritual needs whilst recognising the essential contribution of the living environment and family caring to the wellbeing of the person with advanced dementia. *Palliare* is a best practice approach to delivering advanced dementia care with dignity and compassion led by professionals with the appropriate specialist knowledge and competencies, and whenever possible in partnership with the individual, their family and friends. There appears to be synergy with the principles of Prudent Healthcare and emerging descriptions of dementia *Palliare*. The discussion acknowledges that the experience of family carers and the capacity to sustain family caring influences the development of plans for care and decisions about place of care and as such family caring needs to connect with considerations of prudent advanced dementia care. This paper draws on various evidence including the policy related experiences and comparisons made by the seven European countries who were partners in the recently completed European Dementia *Palliare* Project (2014–2016). The *Palliare* partnership comprised interdisciplinary teams from Scotland UK, Czech Republic, Finland, Portugal, Slovenia, Spain and Sweden, full details and project outputs are available at [7].

In all countries the aspirational nature of policy documents sets future direction and policy: implementation though follows at a variable pace. The findings of the *Palliare* project, which focussed on equipping the European dementia workforce to champion evidence informed improvements in advanced dementia care, suggests that it is possible and highly desirable to align policy innovations with new integrated practice models and to accelerate implementation through enabling inter-professional learning frameworks. Furthermore from a policy perspective, the utility and impact of the Prudent Healthcare paradigm in advanced dementia care will rest on a national policy and service delivery commitment to embrace the key prudent principle of ‘provide the best possible care for those with the greatest healthcare needs first’. Explication of principles underpinning policy and policy decisions is essential for transparency, consistency and values alignment arguably eases the route to policy implementation. The appeal of the Prudent Healthcare model is a mix of clarity of the underlying intentions and the wise use of limited resources; however an inherent risk in advanced dementia care is that prudency might inadvertently serve to perpetuate the ‘inverse care law’ where those who need most care receive least. We examine the advantages and disadvantages of the prudent perspective as a potential lens for policy making related to reforming advanced dementia care in seven European countries.

## Prudent Healthcare Paradigm

The espoused development imperative across all health and social care is for good quality, safe and efficient, person-centred practice delivered with compassion. While no one can argue against this quest, economic pressures and inequalities in provision mean that existing care systems cannot simply expand to meet the challenge of increasing numbers of people, coupled with the ageing demographic of the developed world. Transformational change is required to meet this challenge, a change in which everyone takes responsibility for health and wellbeing in a united and integrated way. Prudent Healthcare is one such type of transformational change and is defined by Aylward et al [5] as:

“healthcare which is conceived, managed and delivered in a cautious and wise way, characterized by forethought, vigilance and careful budgeting which achieves tangible benefits and quality outcomes for patients.”

Prudent Healthcare has been influential in a number of healthcare system reforms [8,9,10,11]. Metzelthin, Daniela, van Rossum et al [9] and de Block [10] are examples of where the principles of Prudent Healthcare have been used to transform discreet services. Collins [11] describes a whole-system transformation of health services in Alaska (Southcentral Foundation’s ‘Nuka’ system of care). This involved integration of primary, secondary and tertiary healthcare services as part of a service redesign, including consultation with the local community. ‘The Nuka’ system reports lower costs and better outcomes and ascribes some of its success to the investment in workforce training. Berwick [12] highlights some of the changes in thinking and leadership that are required to bring about such transformations: using measurement for learning, not judgements; aiming for continual improvement, everywhere; respecting and engaging the workforce as valuable contributors of new ideas; and, vigorously pursuing the needs and experiences of service users. Loeffler and colleagues [13] preceded this thinking by having co-production as a key strand of service redesign; this can greatly enhance the principles of Prudent Healthcare by recognizing and utilizing the contribution that service users can make to the commissioning, planning, design, prioritisation, financing, delivery and management of the services they use. Co-production is described as:

“professionals and citizens making better use of each other’s assets, resources and contributions to achieve better outcomes or improved efficiency” (15:23)

Accordingly Aylward et al [5] describe the cornerstones of Prudent Healthcare as:

- Healthcare that fits with individual needs and circumstances
- Approaches that avoid waste and harm
- Avoidance of interventions that bring no or little benefit
- Optimal and fair use of resources.

In other words, prudent approaches claim to deliver individualized care, with judicious decision making to make the most of available resources, and avoidance of care which does not bring benefit, or is not wanted, or is not beneficent. By embracing this value base the promoter of the prudent paradigm rejects notions of rationing. Importantly as Bogdan-Lovis & Holmes-Rovner [14] argue, the prudent perspective has much to offer evidence based medicine through promotion of shared decision making and patient choice.

The scale of reform to achieve quality dementia care also requires careful decision making and prudence. The benefits of the prudent collaborative stance in dementia care are exemplified in the Dutch Geriant Model of integrated community-based dementia care [15]. Mutlidisciplinary practice delivered within integrated services are at the heart of the Dutch model which is described as responsive to the progressive and changing needs of people with dementia. So in theory and in practice it seems that the prudent perspective has potential to assist us to tackle ‘the inverse care law’ and address the inequality that exists when those in most need of care receive the least services and inadequate support [5]. This rebalancing of care to those with greatest need is central to improving services and the *Palliare* practice improvement imperative.

Furthermore in many countries dementia care is afforded low status, there are workforce recruitment and retention challenges and gaps in practitioner education. The report, ‘A Call for Action’ [16] states that workforce training is essential to build capacity and to relieve the burden placed on family carers of people living with dementia. It highlights that the workforce includes all professionals who might interact with the person with dementia from pre-diagnosis through to end-of-life care, and that any education must take cognizance of local health and social care systems. The authors provide an example of good practice from China [17]. This is a training programme available to both professional and family caregivers which promotes person-centred care and aims to develop a better understanding of the behaviours associated with dementia, therefore enabling the provision of more effective and compassionate care. Another Prudent Healthcare concept is “do only what only you can do”. This means that for the future workforce, no one should be routinely providing services which do not require their level of ability or expertise.

## Advanced Dementia Practice, Education and Policy

Dementia is a progressive condition associated with chemical and structural changes in the brain caused by illnesses such as Alzheimer ’s disease. In the early stages people may be able to live independently. As the disease progresses, living well with dementia requires increasing levels of support and care. The middle to late phases of the disease often signal a loss of autonomy, independence, physical and cognitive function. This phases is the beginning of an extended palliative care phase [18]. Van der Steen et al [19] make a compelling case for dementia appropriate palliative care.

The European Dementia *Palliare* Project has responded to the challenge to develop a response to improve dementia care and in particular to improve approaches to advanced dementia care through providing educational solutions to a workforce that deliver services, care and support across health and social care. Importantly a distinction was made between the dying phase and dying, and the phase prior to this which is about living with advanced dementia. The objective of the first phase of the *Palliare* project was to develop an inter-professional understanding of best practice for advanced dementia care and family caring, and an understanding of the contribution of different disciplines to the achievement of best practice. The second phase objective was to develop an innovative virtual inter-professional experiential learning and resources to equip the European qualified dementia workforce to transform advanced dementia care and deliver best practice. This paper draws on the *Palliare* project findings including a policy review, a literature review, experienced based case studies, and education gap analysis all of which informed the development of a European Dementia *Palliare* Best Practice Statement [6].

## Overview Methods

The Policy Review was completed during 2014 and involved desk based research using a proforma to identify and retrieve relevant policy documents from each of the seven partner countries, in addition to online country policy reviews prepared by Alzheimer Europe and Alzheimer Disease International [22,23]. Country based teams reviewed their own policy documents and extracted sections relevant to dementia palliative care, integrated services and dementia workforce development. Teams initially worked in their own language and prepared English Language summaries to facilitate comparative analysis.

An integrative review methodology [20] was used to review the literature that discussed the experience of dementia associated with the extended palliative phase. The aim was to explore how this experience was described, what was known about symptomatology and management and the needs and preferences of the person with dementia and their family. A standard search and appraisal protocol was applied by review teams which yielded an initial of pool of 492 papers, 109 of these papers where included in the review [21].

Qualitative case studies were guided by a research protocol centred on the experiences of the person with dementia and their family [24]. Local research teams were responsible for securing the ethical and management permissions to research with this vulnerable group of people. Mindful of the country specific requirements and contextual variation the case study protocol was adjusted as required to permit the building of cases to generate meaningful understandings about giving and receiving of care from the perspective of the individual, family and involved practitioners. A total of 22 in-depth case studies were constructed using open questioning approaches with the case study informants. Preliminary simple content analysis was undertaken by local teams to code and categorise data and findings were compared across their own cases and the full set of 22 cases. The second level of analysis was undertaken by one team working on English translations to achieve cross case comparisons to reveal the unique and shared experience across the data set.

The educational gap analysis used a combination of desk based research supported by discussions led by local team members with key informants, mainly education providers [25]. A list of relevant educational provision, including continuing professional development courses through to degree programmes was generated for each of the seven countries. The learning outcomes from these training and education programmes were mapped against the evidence based *Palliare* Best Practice Statement [6], which had been endorsed through external consultation with national agencies, practitioner communities, Alzheimer Associations including Alzheimer Europe.

## Interpretation of Findings

It is widely believed that a national dementia plan is a catalyst to improving dementia care. In 2013 only 11 countries worldwide had developed or implemented national dementia plans. Eleven of the 28 member countries of the European Union have national dementia plans which address dementia awareness raising and education, diagnosis and treatment, home and institutional and residential care [22]. Pot and Petrea [23] highlight the importance of including end-of-life care and palliation within national dementia plans. However, as Piers et al [26] reported, few European countries routinely offer specialist palliative care to older people with non-malignant disease, and fewer people with dementia have access to dementia specific palliative care services despite this being a recognised need [27]. The *Palliare* Policy Review completed with seven countries revealed that Finland and Scotland had the most established and comprehensive national dementia action plans, and that in three countries (Czech Republic [28], Slovenia [29] and Portugal [30] national dementia action plans were in development. Interestingly the definition of the third stage of dementia within the strategy of the Czech Alzheimer Society (the P-PA-IA) aligns with *Palliare* and embraces integration of health and social care [31].

In Sweden the national strategy on dementia and long term care facilities recommends that staff should have at least nurse or ancillary nurse education with specific skills in dementia [22]. It is also noteworthy that the Slovenian Resolution on the National Social Protection Programme [32] described dementia care in some detail, with an emphasis on the reform of institutional care for a range of clients including people with dementia. The Portuguese Action Plan from the Integrated Continuous Care National Network, tasked a working group (Despacho nº 201/2016) to develop local strategies and responses for palliative care delivered specifically to people living with dementia, and for children. In Spain there is no law that considers specifically, the palliative care of people with advanced dementia, and nor are there any national action plans or strategies related to this population. The Spanish Clinical Practice Guideline is based on healthcare for people with Alzheimer’s disease and other dementias [33] and is the only guideline regarding palliative care in advanced dementia patients and their families in terms of: context definitions, resources, management of clinical situations and support, attention to family caregivers and ethical issues. All seven countries reported existence of some type of national action plan for generic palliative care.

The literature review [21] concluded that healthcare needs should be foregrounded within Dementia *Palliare*, and revealed an evidence base for practice of variable quality. The research literature was dominated with studies concerning the terminal stages of death and dying, with less attention to interventions and care designed to help people with advanced dementia to live well. This led to a recommendation for a new narrative and move towards advanced dementia specific and positive approaches to the extended period of living prior to the terminal stages of dementia and end stage palliation, hence the call for dementia *Palliare*. Only one advanced practice model was located from Scotland, this as yet untested model presents an integrated approach which includes the creation of Advanced Dementia Specialist Teams [34]. While this has been broadly welcomed in social media and is evidence based, its utility in practice, uptake and associated costs are not yet known.

22 Case Studies developed as part of the Palliare project revealed that fragmentation of care and limited choices to support individuals with advanced dementia and their caregivers was common. Not unsurprisingly experiences which created a sense of continuity, with timely access to experts, respected the preferences of the individual and enabled family caring were described more positively than those which felt discontinuous and lacking in expert input [24]. The case study method was a useful strategy for the investigation of such a complex phenomenon due to the different contexts’ characteristics and cultures explored [35]. Early diagnosis, good coordination between service providers, future planning, support and education for carers, enabling the person with dementia to live the best life possible and education on advanced dementia for professional and family caregivers were all significant issues considered important for positive care experiences through the cases explored.

The education gap analysis [25] demonstrated limited input on dementia care and even less on advanced dementia care in undergraduate and postgraduate health and social care programmes across the seven partner countries. Many professionals such as nurses, social workers, psychologists and dieticians including those who work in older care settings do not receive specific education on dementia [36,37,38,39,40]. Mustafa et al [41] assert that ‘the numbers of staff who potentially need dementia education and training are huge’ (30:99).

Furthermore dementia workforce development plans with the exception of Scotland’s Promoting Excellence [42] are not embedded in policy and no workforce planning explicitly related to upskilling staff to deliver improvements in advanced dementia practice was located. The case studies [24] and the education gap analysis [25] therefore supported the lack of palliative care education and knowledge of the late stage of dementia which is reported in several studies [43,44,45,46,47,48]. The case studies [24] supported the literature, in that often needs of people with dementia and their families are not met [47,49,50,51,52] care experiences were fragmented and the place of palliative care for people with dementia ranged from the family home, to hospital and care homes.

## Integrated Dementia Care

The review of policies and national action plans illustrated there were some moves to integrated approaches in most of the seven countries. As the integration of health and social care is seen as being a key approach in meeting the principles of Prudent Healthcare we examined the key policies, strategies and guidelines that supported or promoted the development of integrated dementia services within the seven participating countries.

Most had policy directives promoting some level or type of integration between health and social care services. Integration between health, social services and other care providers is one of Scotland’s major programmes of reform. The purpose of this reform is to ensure that people get appropriate care and support throughout their journey of care [53]. In Sweden we see a drive for integration across primary, community, hospital and tertiary services as Sweden’s Health & Medical Services Act [54] para 26e states:

“The county council and municipality shall cooperate so that the individual in the municipality for whom they are responsible according to paragraph 18 also receives other care and treatment, assistive devices & products according to his or her requirements according to paragraph 3d”.

Almost identical to the principles of Prudent Healthcare, the Health Care Act in Finland [55] specifically states the aims of promoting and maintaining the population’s health and welfare; reducing health inequalities; ensuring universal access to services; and, improving quality and patient safety, and strengthening cooperation between care providers. In Finland we also find integration of care within one sector; dementia care is embedded within Mental Health Services specifically within the National Memory Programme [56]. The objective is to build a memory friendly Finland on the basis of four pillars:

1. The promotion of brain health
2. A more open attitude towards brain health, and the treatment and rehabilitation of progressive memory disorders
3. Support, treatment, rehabilitation and services at the right time to promote a good quality of life for people with progressive memory disorders and their families, and
4. Increasing research and education.

The concept of “integrated care” is emerging as a new paradigm in Portugal, with the capacity to provide solutions to many problems created by the division of health and social services. Currently dementia care is embedded in Mental Health services and the overall objective is to integrate Mental Health services into the generic healthcare system. This form of integration aims to promote robust mental health and ensure equity of access for everyone, including those with dementia. Underpinning the decentralisation of mental health services and the move to locally based services is the ambition to enable increased participation of individuals and communities and the adoption of a human rights based approach to care and support [58]. The care provided is based on a joint assessment and seeks the recovery of the whole person, where the person is seen as the centre of a process of continuous therapeutic and social support [57].

The Spanish Government also promotes decentralisation and has allowed its 17 regions to develop different laws and regulations from each other and there is no national legislation apart from the Law of Dependency. None of these laws and regulations is specific to dementia care though they cover end-of-life care, for example in the regions of Andalucía, Aragón and Navarra.

So we see a variation of integration themes, models and methods. As can be seen from the *Palliare* policy review [59] and recent literature [60] the Prudent Healthcare principle of reducing inappropriate variation using evidence based practices is central to extant dementia policy and policy in development. All the current and emergent plans acknowledge the need for further research, redesign of services to ensure best use of resources and for improved education for formal caregivers in health and social care, it is to this we turn next.

## Workforce and Skill Mix for *Palliare*

In response to calls for countries to develop national actions plans and for common guidelines to inform practitioner training [3] as previously stated, the Dementia *Palliare* Project has ambitions to support the development of expert advanced dementia practice and integrated dementia care. Education of the wider healthcare team to deliver palliative care to older people with dementia also featured in the recommendations of the recent European Association of Palliative Care White Paper [61]. Thus not only do dementia practitioners need to understand how to support people to live well with advanced dementia and to plan for palliation, but palliative care experts are encouraged to develop skills to support people with advanced and end stage dementia.

Education delivered in an accessible and interactive way would help to improve the status of care workers, and assist in relieving the burden on family carers. Family carers of people living with dementia are presently supported by a range of health and social care professionals, and this skill mix differs across countries. Similarly, the education and training of professionals varied across the partner countries. Whilst all countries had education and training available for palliative care, Scotland and Sweden were the only countries where this specifically addressed the palliative care needs of people with dementia and their families. Thus innovative inter-professional experiential learning would support the development of an integrated workforce through the development of shared values and language, in addition to knowledge.

## Discussion and Conclusion

As initially argued in the introduction to this paper, Prudent Healthcare seeks the wise use of limited resources shaped around guiding principles that promote partnerships between the providers and receivers of care. The current drive for integrated practice and services rests on the quest for efficiency and continuity of person centred improvements. Superficially there appears to be coherence between the two approaches and as such both perspectives offer useful ways to progress policy directions reshape services and guide new practice models to improve advanced dementia care. Partnerships with patients and listening to what people want (including family) from healthcare is a feature in the prudent approach. Alzheimer associations across Europe and Family Carer networks consistently argue for policy makers and service providers to listen to the concerns and experiences of people with dementia so that co-production is a key strand of service design and redesign. The emphasis on co-production and partnerships between practitioners and the public is clearly welcomed and exemplified in the prudent collaborative stance taken in the Dutch Geriant model of integrated dementia community care [15]. However, as an individual’s condition advances and their ability to express their own views is compromised, sustaining true partnerships with the individual is likely to become increasingly difficult. Notwithstanding this difficulty the Glasgow Declaration acknowledges an individual’s right to person centred, coordinated, quality care throughout their illness [62].

This highlights the importance of strategies to capture in advance the wishes of the person and give others an understanding of the person to help us to plan how we can sustain person centred approaches. If we fail to plan for advanced dementia care and passively accept over reliance on the proxy view this will undermine the principle of co-production.

Our discussion has also recognized an inherent risk that in advanced dementia care prudency might inadvertently serve to perpetuate the ‘inverse care law’ where those who need the most care receive the least. Furthermore we argue that for integrated services to deliver high quality care to people with advanced dementia that practitioners need to understand best evidence informed advanced dementia practice, and be equipped with knowledge and skills and be empowered to deliver it within often impoverished care environments. Impoverished care environments have been described as environments where professionals, carers and cared for people do not routinely feel a sense of security, belonging, continuity, purpose, achievement and significance [63]. Creating enriched as opposed to impoverished care environments for many service providers is a challenging quest.

The evidence reviewed within this paper reveals a number of other challenges for European administrations. The absence of clear agreement and definition about advanced dementia, and a lack of attention to dementia specific palliative approaches are two of note. Mapping the experience of Advanced Dementia Care in Europe as developed through the Dementia *Palliare* Project Case studies [24] provided one approach to the issue by directly investigating care practices in real and typical settings with the participation of people with advanced dementia and their carers. The differences in the advanced dementia care experience amongst the seven European countries participating in the *Palliare* Project, consistently revealed the need for improvement in the services that are provided for people with advanced dementia within the family home, hospitals and care homes. The 22 cases explored confirmed that the individual and family experience of our European services and practices were varied and fell short of both public and professional expectations given the available evidence base. In part this was explained by the absence of a practice model which was needed alongside enlightened national dementia policies.

The preparation of a European *Palliare* Best Practice Statement [6] which focusses on living the best life possible with advanced dementia goes some way to address this lack of clarity and adds detail missing from the progressive dementia care goals models proposed by van der Steen et al [61]. The Best Practice Statement [*6*] however, is a tool to guide and address educational deficiencies in interprofessional dementia education based on currently available evidence. Its utility in terms of policy and practice is yet to be tested, although it has explicitly influenced a newly proposed Advanced Dementia Practice Model within Scotland [34]. Once the Best Practice Statement [6] has been used across Europe, new case studies will be needed in the same European contexts to keep monitoring and evaluating what works well in Dementia Care and also to identify and improve any further negative aspects of care. The international call to action to improve healthcare for people with dementia at the heart of the 2016 World Alzheimer Report recognizes the need for high quality palliative and end of life of care and recognition of dementia as a terminal condition [64]. In responding to this it will be important to appreciate that the need for dementia specific *Palliare* is not confined to the last stage of life, and that the evidence presented herein suggests that individuals and family welcome an approach that embraces living, not just end of life. Furthermore for people to experience a sense of living the best life possible with advanced dementia there needs to be confidence in and quality of healthcare provision delivered within an integrated approach.

Turning to the literature concerning other advanced long term conditions, there is also a clear consensus about the appropriateness of palliative care (see for example for people with advanced chronic heart failure (CHF) and chronic obstructive lung disease (COPD) [65]; advanced chronic kidney disease [66]; progressive neurological disease [67]; Parkinson’s disease [68]. In particular, there is widespread recognition of the urgent need to integrate palliative care in the everyday care of patients with advanced, long term chronic conditions. However, a recent systematic review [65] of European guidelines and pathways on the integration of palliative care in patients with CHF and COPD reflects a growing realization that in reality this simply is not yet happening on a routine large scale [68,69,70,71]. Service delivery models are predominantly still at the project stage [72,73,74,75]. Common to these initiatives and similarly to *Palliare*, is the importance of service delivery models that focus on a team based approach and an emphasis on interlevel dynamics at individual, team, interdepartmental and organizational levels. Likewise, there is a strong focus on palliative care education for all professionals involved in care of patients living with advanced, long- term conditions and their families. Thus, the broader debate that is currently occurring in the delivery of palliative care across advanced, long term conditions clearly resonates with the concept of *Palliare* and a Prudent Health care perspective.

In conclusion, the Prudent Healthcare perspective can give useful structure to debates about principles to underpin reform of advanced dementia care. By starting with questions about what people with dementia and family carers want it may be possible to imagine new integrated services within the family home, and housing alternatives with integrated care. Such a new starting point might be more helpful than simply trying to make small scale changes or repairs to what already exists but is known to be inadequate. Adoption of the prudent perspective will however require development of a repertoire of approaches to hear the voice or proxy voice of the person with advanced dementia, and commitment to develop and implement new evidence underpinning prudent dementia *Palliare*. For many this would be a useful place to start reform.
